# Random forest-based bioavailable strontium isoscape for environmental and archaeological applications in central eastern Argentina and western Uruguay

**DOI:** 10.1371/journal.pone.0326047

**Published:** 2025-07-15

**Authors:** Cinzia Scaggion, Tommaso Giovanardi, Daniel Loponte, Mirian Carbonera, Elena Armaroli, Sara Bernardini, Stefano Benazzi, Andrés Gascue, Alejandro Acosta, Giulia Marciani, Eugenio Bortolini, Anna Cipriani, Federico Lugli

**Affiliations:** 1 Department of Chemical and Geological Sciences, University of Modena and Reggio Emilia, Modena, Italy; 2 Consejo Nacional de Investigaciones Científicas y Técnicas (CONICET), Instituto Nacional de Antropología y Pensamiento Latinoamericano (INAPL), Buenos Aires, Argentina; 3 Programa de Pós-graduação em Ciências Ambientais e Centro de Memória do Oeste de Santa Catarina da Universidade Comunitária da Região de Chapecó (Unochapecó), Chapecó, Brasil; 4 Department of Cultural Heritage, University of Bologna, Bologna, Italy; 5 Universidad de la República (Uruguay)-Centro Universitario Regional del Este -Departamento de Sistemas Agrarios, y Paisajes Culturales, Rocha, Uruguay; 6 Lamont-Doherty Earth Observatory of Columbia University, Palisades, New York, United States of America; 7 Institut für Geowissenschaften, Goethe-Universität Frankfurt, Frankfurt am Main, Germany; Khalifa University, UNITED ARAB EMIRATES

## Abstract

Bioavailable strontium (Sr) isoscapes are essential tools in studies on environmental processes, animal and human mobility and provenance. The success of these studies relies on the comparison between the measured ^87^Sr/^86^Sr isotope ratios of specimens and the spatial distribution of environmental bioavailable Sr isotopic signatures across geographical regions. A critical step of this process is the construction of reference maps that integrate environmental Sr isotopic data with geographical information. Here, we present a new bioavailable Sr dataset of 113 environmental samples, including plants and malacological samples collected from center-east Argentina (Paraná Delta, Pampa and Entre Ríos plains) as well as adjacent Uruguay, covering an area of approximately 122,500 km^2^. This dataset is further integrated with archaeological bioapatite data from the literature to construct the first random forest-based Sr isoscape of the region. Notably this area is on recent Quaternary (fluvial, marine and aeolian) sediments derived from the erosion of magmatic and metamorphic terrains with different Sr isotope composition from low ^87^Sr/^86^Sr ratios (about 0.706) to highly radiogenic signatures (>0.71), and heterogeneously transported in the Delta area by the rivers and in the high plains by wind and rivers. This isoscape offers a unique perspective on the Sr isotope distribution in a lithologically homogeneous region characterized by relatively young sedimentary sequences. This work represents a significant advancement in the development of Sr isoscapes, providing a fundamental tool for environmental and archaeological applications in South America.

## Introduction

Isotopic maps of the landscape, isoscapes, represent powerful tools for understanding the spatial distribution of isotopic signatures in both modern and ancient contexts. These maps have proven instrumental in addressing questions related to migration, trade, diet, provenance, forensic and environmental change [1–15].

Strontium isotope ratios are particularly valuable in these studies, as they provide insights into the geological and environmental histories of regions and their influence on biological systems. They are relatively stable over time (49.61 ± 0.16 Ga for the half-life of ^87^Rb; [16]) and therefore act as geochemical tracers to determine the geographic provenance of modern materials, ancient artifacts, as well as fauna and human mobility, identifying non-local individuals [11,12,14].

Unlike other isotopic systems (e.g., hydrogen, oxygen, carbon, nitrogen isotopic systems), where geolocation information is limited due to their large gradients on the Earth’s surface and the difficulty of predicting spatiotemporal isotopic variations, the ^87^Sr/^86^Sr ratio follows defined geological regimes and a predictable spatiotemporal variability at high resolution [17,18]. The evolution of the radiogenic isotopic ratio of strontium depends mainly on the radioactive β-decay of ^87^Rb to the radionuclide ^87^Sr thus lithologies, geological histories, ages and original ^87^Rb contents result in different abundances of ^87^Sr in rocks [19,20]. The local bioavailable ^87^Sr/^86^Sr ratio is reflected in organisms that have fixed local strontium in their tissues, as a sort of *isotopic fingerprint* of a given geologic province.

Strontium in water bodies originates from the leaching of local bedrock, as well as from additional sources such as rainfall, snow, groundwater, and atmospheric deposition [21,22], which can carry the isotopic signatures of non-local geological formations. Sr released into the environment becomes bioavailable [23] by entering in the local ecosystem through soil and water [24]. For this reason, the signal of the bioavailable strontium pool may often be different from that of the local bedrocks. This bioavailable strontium (as cation Sr^2+^) is taken up by plants in substitution of calcium (Ca^2+^) [25] and is passively absorbed in the root system and leaves replacing Ca essential for metabolic processes [26]. From plants and water, the Sr enters the trophic chain replacing Ca into the crystalline structure of carbonated hydroxylapatite of vertebrates [27,28], a biomineral [29] that mainly constitutes dental and bone tissue [30,31]. The ^87^Sr/^86^Sr ratio in bone tissue should thus reflect that of consumed water, plants and animals, which in turn reflects the bioavailable isotopic signatures of the catchment in which individuals lived [32,33]. While strontium isotope fractionation can occur during trophic transfer and uptake into skeletal tissues, the effect is minimal and generally undetectable with standard analytical instruments [34]. Any slight fractionation is corrected through standard normalization protocols, ensuring that the Sr isotope composition in bone and enamel accurately reflects environmental sources [24,35]. The bioavailability and assimilation of Sr in the skeletal system are governed by a complex interplay of geological context, biological processes, and dietary habits. Several environmental samples (such as plants, soil, and low-mobility animals) and several modelling approaches (e.g., domain and contour models, geostatistical mapping, and machine learning approaches) have been employed in the building of strontium large-scale isoscapes [36–39] for a complete overview see [40].

Recently, machine learning methods such as random forest regression [15,18,40–42] have significantly improved the predictive power of isoscapes by combining ^87^Sr/^86^Sr data obtained from strontium isotope data sources with those of environmental auxiliary predictors, even at global scale [43]. However, this type of statistical approach is not free from uncertainties related to the use of various isotope data repositories and therefore a targeted primary analysis of valid datasets (e.g., plants) collected in pristine landscapes is generally recommended to increase model accuracy by local characterization of bioavailable Sr [43].

The Paraná Delta region (central-eastern Argentina) and its adjacent areas (the Pampa and Entre Ríos plains, and southern-western Uruguay) represent an interesting zone not only for provenance studies (for both archaeological questions and modern exportation), but also to define the role of hydro-geomorphological and sedimentological processes in the formation of floodplains [44]. In this region, isotopic studies are mainly focused on archaeological and environmental research, producing data of stable isotopes [45–52]. Conversely, Sr isotope data are very few (i.e., bone tissue in [49] and fluvial waters in [53,54]), highlighting the need of novel reference values for the area. The work proposed here aims to evaluate the dominant environmental and geological controls of bioavailable ^87^Sr/^86^Sr and predict isotopic variation in the survey area to build a large-scale high-resolution isoscape useful for environmental, provenance and mobility studies. For this purpose, we build a new dataset of ^87^Sr/^86^Sr isotopic ratio from plants (109 samples) and snail shells (4 samples) from the study area which cover c.a. 122,500 km^2^. Data were modelled through a random forest algorithm to build a first bioavailable ^87^Sr/^86^Sr isoscape of the region, incorporating geological, climatic and environmental covariates. A challenge for the model is the relatively homogeneous lithologies outcropping in the studied area, being mainly sediments (silts or clays) of recent age (Quaternary) but related to several different geological context (fluvial, coastal, marine and continental) which in large scale geological maps are grouped in a single unit [55,56].

### Regional geology

**Fig 1:**
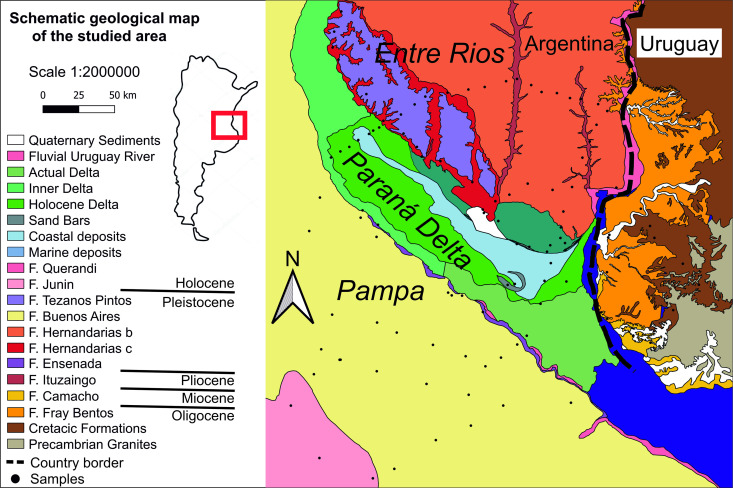
Spatial distribution of geological units. Geological sketch map of the studied area based on the available geological map of Bertolini (1995; Mapa Geologico de la Provincia de Entre Ríos, 1:500000), Rimoldi (1999; Mapa Geologico y Minero de la Provincia de Buenos Aires, 1:750000), Pereyra et al. (2005, Hoja Geologica 3360IV, Gualeguaychù, 1:250000, 2) and Loureiro Olivet et al. (2024, Actualización de la Carta Geológica del Uruguay a escala 1:500.000, Ministerio De Industria, Energía Y Minería, Dirección Nacional De Minería Y Geología) [55,56,59,62]. Quaternary Sediments, Fluvial Uruguay River, Actual Delta, Inner Delta, Holocene Delta Sand Bars, Coastal Deposits, F. Querandi and F. Junin are Holocene. F. Tezanos Pintos, F. Buenos Aires, F. Hernandarias and F. Ensenada are Pleistocene. F. Ituzaingo is Pliocene. F. Camacho is Miocene. F. Fray Bentos is Oligocene.

The Paraná Delta is a depressed wetland extending approximately 320 km northwest of Buenos Aires, with variable width. It is separated by the adjacent Pampa and Entre Ríos plains by steep cliffs. This region is both a complex estuary delta [57] and a wetland, influenced by water tides [58]. The surface geology is composed of Quaternary sediments related to fluvial, delta and marine environments [59,60]. During the middle Holocene, marine ingressions deposited sands and clays from Southeast to Northwest. The end of the ingression and the advance of the delta covered the marine geological units with fluvial sands and clays in the upper and southern parts of the Delta, extending to the cliff bordering the Pampa plains [59]. Currently, marine sediments outcrop mainly in the lower part of the Delta up to the cliff with the Entre Ríos plain. The end of the wetland bordered by the Uruguay River and the ocean is occupied by delta sediments (Fig 1). Both marine and Paraná sediments are locally covered or interbedded with silts and subordinate sands deposited by tributaries from the Pampa and Entre Ríos plains (e.g., Gualeguay, Arrecifes, and Nogoya rivers among others).

The cliffs separating the depressed wetland from the adjacent plains mainly expose Pleistocene horizontal units of the Ensenada and Buenos Aires formations, formed by silt loess deposits [59]. Within the Pampa plains, two formations outcrop on the surface: the Pleistocene Buenos Aires Formation, consisting of homogeneous loessic clays with calcareous nodules, and the Holocene Junín Formation, characterized by loessic sands [56] (Fig 1). Within the Entre Ríos plain, the main lithology is the clay of the Middle Pleistocene Hernandarias Formation (Fig 1). These clays are divided in two main subunits: the Hernandarias Formation *c*, outcropping in the North and the East formed by playa sediments of the Uruguay River, and the Hernandarias Formation *b*, formed by loess deposits [55,60,61]. The latter is covered by the Upper Pleistocene loess deposits of the Tezanos Pintos Formation, at the border with the upper and central part of the wetland [55]. Erosion by the Gualeguay and Gualeguaychú rivers has exposed lower sands of the Lower Pleistocene-Pliocene Ituzaingó Formation [55] (Fig 1). At the eastern border of the Entre Ríos plain, erosion by the Uruguay River exposes the sedimentary stratigraphic sequence down to the Oligocene Fray Bentos Formation silts.

The Fray Bentos Formation mainly outcrops east of the Uruguay River, within the Republic of Uruguay (Fig 1). It is in discordant contact with the Upper Cretaceous sandstones of the Asencio Formation and the Mercedes Formation, deposited in a continental arid environment [62]. Neogene and Quaternary climate change and marine ingressions/transgressions have shaped the sedimentary record near the Uruguay River [62]. The sandstones and sands of the Miocene Camacho Formation, the Pleistocene Chuy Formation and the Holocene Villa Soriano Formation are related to marine environment (Fig 1). Conversely, the Pliocene-Pleistocene sandstones and sand deposits of Raigón, Salto (the counterpart of the Ituzaingó Formation in Argentina) and Paso del Puerto Formations, as well as mudstones and silt-clay sediments of Libertad and Dolores Formations are related to fluvial deposition [62].

## Materials and methods

### Samples collected and literature

**Fig 2:**
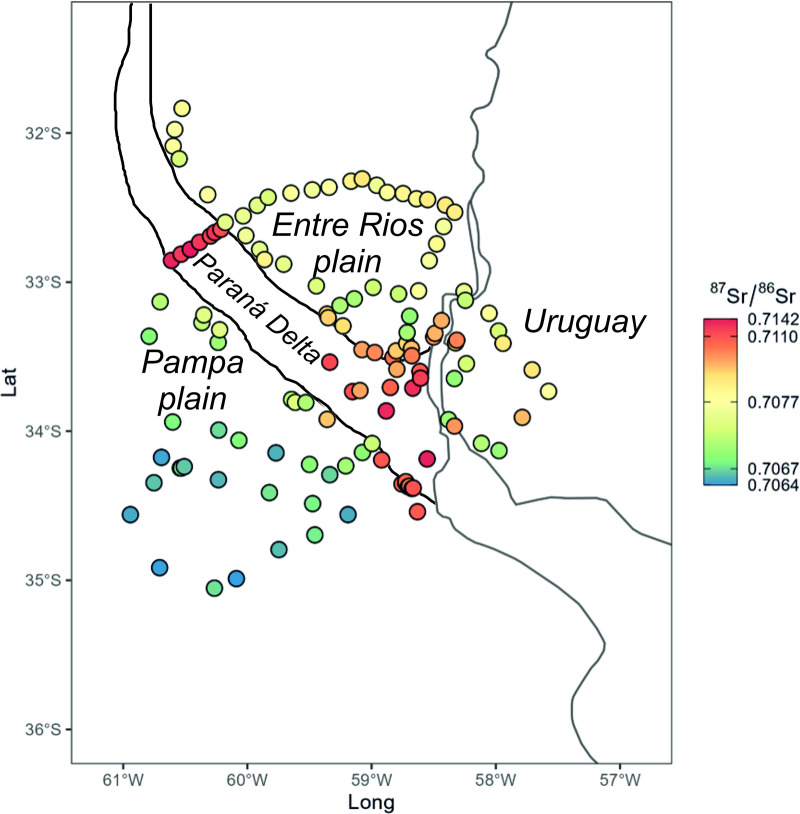
Strontium isotope distribution results. Geographical distribution of new and literature samples in the studied area coloured based on ^87^Sr/^86^Sr values.

Currently, only 4 bioavailable Sr isotope data points are reported for the study area in the literature and they are river waters of the Paraná Delta [53]. To expand this dataset, a total of n = 4 snail shells and n = 109 medium/deep- rooted plant samples were collected from different geological units across the Paraná Delta and its adjacent regions. These geological units were mapped using the Sagemar geological cartography (Mapa Geológico y Minero de la Provincia de Buenos Aires, 1:750000, Rimoldi, 1999; Mapa Geológico de la Provincia de Entre Ríos, 1:500000, Bertolini, 1995; Hoja Geologica 3360IV, Gualeguaychú, 1:250000, Pereyra et al., 2002) [55,56,59]. The study area also includes a small portion of Uruguay located across the Uruguay River (Actualización de la Carta Geológica de Uruguay a escala 1:500.000, Ministerio De Industria, Energía y Minería, Dirección Nacional De Minería y Geología, Loureiro Olivet et al., 2024) [62]. The list of samples with geographical coordinates is reported in the S1 File. The geographical distribution of new and literature data from the area is also reported in Fig 2.

Plants are currently considered among the most accurate environmental indicators for constructing Sr isoscapes [63] because, in addition to being at the base of the food chain and thus representing the bioavailable isotopic fraction [64], they are readily available and ubiquitous across the landscape. However, they require a careful evaluation before being used as a Sr proxy (see, e.g., Frei et al. (2022) [65] and Johnson et al. (2022) [66]). The plant taxa collected from the region include *Eucalyptus*, *Phytolacca dioica*, *Neltume nigra*, *Smilax campestris*, *Celtis australis*, *Fraxinus excelsior* (Common ash), *Nerium oleander* and *Vachellia caven* (Espinillo tree).

An essential step in the plant sampling strategy to obtain the ‘true’ bioavailable strontium isotope signature is avoiding contamination from watercourses, fertilizers, and pesticides related to the effects of modern agriculture [63]. For this reason, samples were collected from trees as far as possible from anthropic activities on the main geological units of the area [66]. Therefore, our sampling strategy focused on covering all local lithological units, selecting several plant species (mainly tree leaves) with different rooting depths, root morphologies in different soils [25].

Snail shells were collected together with onsite plants.

In addition, our dataset includes literature bioavailable Sr data from 56 osteoarchaeological samples, detailed by Loponte et al., (2025) [67] reported in S1 File. The precision and accuracy of strontium data, along with methodological details, including international standards and assessments of diagenetic contamination in archaeological specimens, are thoroughly illustrated in the original publication.

To demonstrate the practical application of our model, we also analysed two archaeological human teeth from the Guaraní site of Arroyo Fredes (within the Paraná Delta) were also analysed. The samples came from two different individuals: a permanent inferior canine from an adult male (AFE-3), aged 20–30 years, and a deciduous upper first molar from a subadult (AFE-4), aged 3.5 years.

### Laboratory methods

#### Sample preparation and extraction chromatography.

The surface of snail shell and tooth enamel specimens was mechanically cleaned and sampled using a low-speed drill, equipped with a round abrasive tip with a diameter of 2.4 mm. Plant samples were cleaned by ultrapure water MilliQ^®^ removing major impurities and dried for 24 h on a hotplate at 50°C. About 10–20 g of dried clean plant material was reduced to ash in a porcelain crucible in a muffle oven at 650 °C for 6 h.

The whole wet-chemistry procedure was conducted in the class-1000 clean-room at the DSCG-UniMoRe. About 5 mg of plant ashes, enamel and snail shell powder were digested in 0.5 ml of 3M HNO_3_ for chemical dissolution. Strontium isolation was performed in 30 μl columns filled with the 100–150 μm bead size Eichrom Sr-spec Resin (Eichrom Technologies, LLC) [68]. After dissolution, the sample solutions were centrifuged and loaded onto clean resin-filled columns. After three steps with 3M HNO_3_ to remove unwanted cations, the Sr was eluted with ultrapure deionized water and collected in clean 15 mL Falcon^®^ tubes. All ﬁnal solutions containing Sr were diluted to 4% HNO_3_ and measured by multi-collector inductively coupled plasma mass spectrometer (MC-ICPMS, ThermoFisher Scientific, Neptune™) at the Centro Interdipartimentale Grandi Strumenti (CIGS) of UniMoRe in static mode, following the methods detailed in Lugli et al. (2020) [69]. Data reduction was performed using the SrDr excel software of Lugli et al. (2020) [69]. Data are reported in S1 File.

Repeated measurements of the strontium isotope reference material NBS987 resulted in a mean ^87^Sr/^86^Sr value of 0.710260 ± 0.000026 (2SD, n = 53). Samples values were corrected for mass bias and normalized to an accepted NBS-987 value of 0.710248 [70]. Reference material data are reported in S2 File by analytical session.

#### Statistical analysis and geospatial modelling.

The local Sr isoscape was generated using the entire dataset (n = 113, of which n = 109 are plants and n = 4 are snail shells) and the associated geospatial coordinates together with archaeological low-mobility fauna dataset from literature [67]. The data were imported into the free statistical software R (version 4.0.5, available at R core Team 2024) and analysed using a Random Forest (RF) machine-learning model described by Bataille et al. (2020) [43]. This method leverages multiple external predictors to model the Sr isotope ratio at 1 km^2^ resolution. The RF algorithm generates multiple decision trees from bagged data, using each time a random subset of data and covariates. In this work, we exploited n = 24 global variables presented in Bataille et al. (2018) [18], Bataille et al. (2020) [43] and Reich et al. (2024) [71] as external predictors of the ^87^Sr/^86^Sr ratio. To avoid multicollinearity in model building, we filtered these variables discarding highly correlated predictors (−0.80 > r > 0.80; S1 Fig.), which could artificially inflate their importance [72]). We ultimately identified a list of (n = 15) external predictors: *r.elevation, r.bulk, r.fert, r.dust, r.pet, r.cec, r.mat, r.bouger, r.meanage_geol, r.ssaw, r.ph, r.srsrq1, r.map, r.clay* and *r.age*.

Random trees were thus built with n = 6 random variables at a time (i.e., optimized *mtry* parameter). To generate a spatial-uncertainty map, we employed a quantile RF regression (*ranger* package, [73]), then halving the RF q_0.84_ ‐ q_0.16_ difference (i.e., lower and upper limits of a ~68% interval: Funck et al., 2021 [74] and Armaroli et al., 2024 [15].

A 10-fold cross-validation was used to determine the model’s performances, evaluated with a root mean squared error (RMSE). This method was selected for its robustness in evaluating model performance, as it involves partitioning the data into multiple subsets (folds) and training/testing the model on these subsets to ensure that the results are not biased by any single subset. This approach provides a comprehensive assessment of the model’s predictive capabilities by evaluating its accuracy and precision across multiple subsets of the data. By dividing the data into several folds and repeatedly training and testing the model on these different subsets, cross-validation ensures that the evaluation is not dependent on a single partition of the data, thereby minimizing potential biases.

## Results and discussion

### Data description and geological inferences

**Fig 3:**
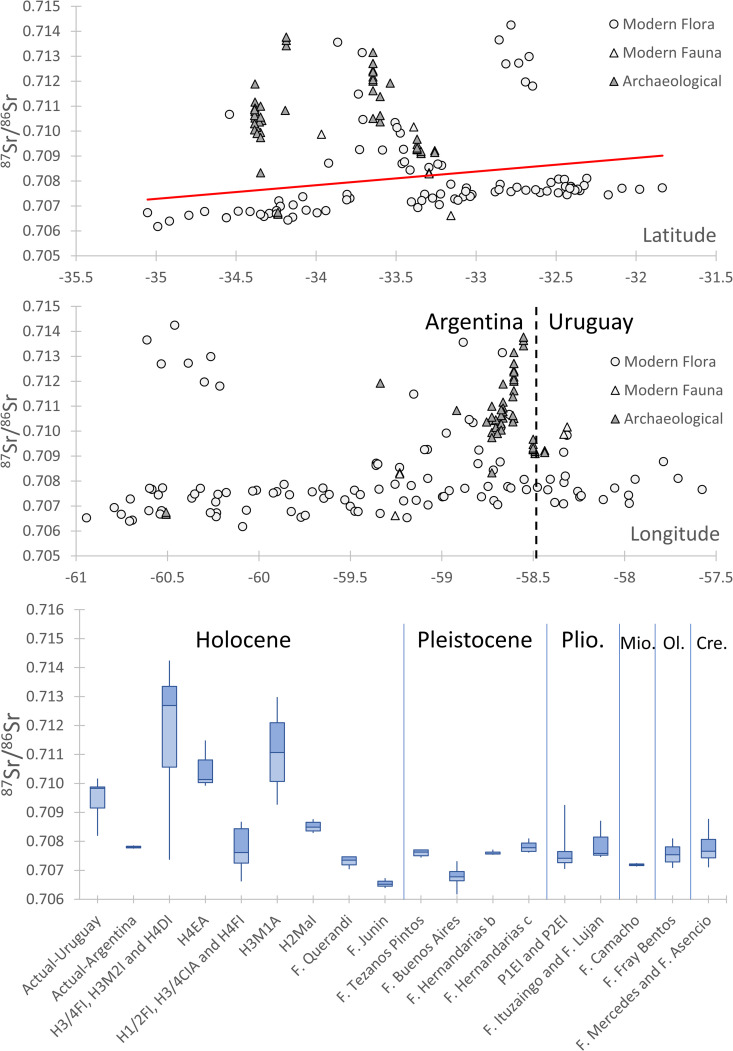
^87^Sr/^86^Sr distribution across latitude, longitude, and geological units. ^87^Sr/^86^Sr values of samples used for the model along latitude and longitude and divided for geological units as in Table 1. Plio. is for Pliocene, Mio. is for Miocene, Ol. is for Oligocene and Cre. is for Cretaceous.

The ^87^Sr/^86^Sr ratios in modern samples from the study area range from 0.70617 to 0.71424 (Fig 3; S1 File). These values envelope those reported in the archaeological record of Loponte et al. (2025) [67], which range from 0.70667 to 0.71377. Notably, the geological units outcropping in the study area are mostly similar in lithology (silts or clays) and age (mainly Quaternary) with distinctions found only in fine-scale geological reports (i.e., the Hoja Geológica 3360IV, Gualeguaychú, 1:250000; Pereyra et al., 2002) [59] mainly related to the sedimentatary environment (i.e., marine, coastal, fluvial or continental). Despite these similarities, the broad ^87^Sr/^86^Sr range found in modern samples indicates that the sources of the sediments and the mixing of different materials plays a more significant role in isotopic variation than lithological or age characteristics of the geological unit. The observed variations in bioavailable Sr isotope ratios are sufficiently pronounced that the resulting Sr isoscape can reliably discriminate among geographic locations within the study area.

**Table 1:** Sr isotopic data by geological formation. Average Sr isotopic data of modern samples collected for this study for main geological formations. The order is based on stratigraphic positions.

Geological unit	Sample number	^87^Sr/^86^Sr average	ST.DEV.	Region	Country	Type of sediments	Age
Actual-Fluvial	5	0.70944	0.00079	Uruguay	Uruguay	Fluvial	Actual
Actual-Fluvial	2	0.70760	0.00008	Pampa and Entre Rios plains	Argentina	Fluvial	Actual
H3/4FI, H3M2I and H4DI	11	0.71179	0.00213	Paraná Delta	Argentina	Fluvial Paraná River	Holocene-Actual
H4EA	3	0.71051	0.00085	Paraná Delta	Argentina	Costal	Holocene-Actual
H1/2FI, H3/4CIA and H4FI	5	0.70773	0.00082	Paraná Delta	Argentina	Fluvial plains rivers	Upper Pleistocene-Actual
H3M1A	4	0.71110	0.00163	Paraná Delta	Argentina	Marine	Holocene
H2MaI	6	0.70851	0.00019	Paraná Delta	Argentina	Marine	Holocene
F. Querandi	4	0.70730	0.00021	Paraná Delta and Pampa	Argentina	Marine	Holocene
F. Junin	3	0.70655	0.00017	Pampa	Argentina	Loessic	Holocene
F. Tezanos Pintos	8	0.70760	0.00012	Entre Rios plain	Argentina	Loessic	Upper Pleistocene
F. Buenos Aires	23	0.70681	0.00029	Pampa	Argentina	Loessic	Upper Pleistocene
F. Hernandarias *b*	13	0.70780	0.00018	Entre Rios plain	Argentina	Fluvial/Coastal	Medium Pleistocene
F. Hernandarias *c*	5	0.70760	0.00007	Entre Rios plain	Argentina	Loessic	Medium Pleistocene
F. Dolores	1	0.70741	0.00002	Uruguay	Uruguay	Fluvial	Pleistocene
P1EI and P2EI	6	0.70768	0.00080	Paraná Delta, Pampa and Entre Rios plains	Argentina	Loessic	Upper Pliocene-Pleistocene
F. Ituzaingo and F. Lujan	3	0.70792	0.00069	Pampa and Entre Rios plains	Argentina	Fluvial	Pliocene-Holocene
F. Camacho	2	0.70719	0.00008	Uruguay	Uruguay	Marine	Miocene
F. Fray Bentos	4	0.70756	0.00044	Entre Rios plain and Uruguay	Uruguay	Continental	Medium-Upper Oligocene
F. Mercedes and F. Asencio	5	0.70781	0.00065	Uruguay	Uruguay	Continental	Upper Cretaceous

The data distribution of modern samples shows consistently higher ^87^Sr/^86^Sr values in the Paraná Delta (average of 0.71070) compared to adjacent areas (average of Pampa 0.70693; Entre Ríos plain of 0.70769; Figs 2 and 3). Exceptions to this in the Paraná Delta include colluvial deposits derived from tributaries crossing the Pampa and Entre Ríos plains (i.e., H1/2FI, H3/4CIA and H4FI units of the geological map from Pereyra et al. 2002 [59]; ^87^Sr/^86^Sr average of 0.70773 ± 0.00082, S.D. n = 5; Table 1, Fig 3) and loess deposits derived from these plains (i.e., P1E1 and P2EI units, average of 0.70768 ± 0.00080, S.D. n = 6; Table 1, Fig 3). Highly radiogenic values in the Paraná Delta are related to the original source of the sediments transported by the river, which crosses for c.a. 4880 km the South America continent. The Paraná in particular flows through the Cretaceous Paraná basin, mainly formed by basaltic and silicic lavas of the Paraná-Etendeka LIP, which exhibit highly radiogenic Sr isotope signatures (e.g., ^87^Sr/^86^Sr of 0.716, [75]).

Among geological units, marine deposits (i.e., H2Mal and H3M1A geological units) have higher ^87^Sr/^86^Sr values than those associated to the plains, with average ^87^Sr/^86^Sr of 0.70955 ± 0.00164 (S.D., n = 10). Since marine sediments near the coast are a mix of continental sediments, their isotopic values fall between the highest and lowest in the region, as the Paraná Delta. Coastal deposits show increased Sr isotope values (i.e., H4EA unit, ^87^Sr/^86^Sr average of 0.71051 ± 0.00085, S.D. n = 3, Table 1, Fig 3), while maximum values are found in Paraná fluvial deposits (i.e., H3/4FI, H3M2I and H4DI units) with average ^87^Sr/^86^Sr of 0.71179 ± 0.00213 (S.D., n = 11, Table 1, Fig 3).

In general, ^87^Sr/^86^Sr values do not show variation along longitude, as would be expected due to the spatial distribution of the geological units, which is mainly NE-SW (Figs 1 and 3). Conversely, a slow-descending trend is observed with increasing latitude (Fig 3).

Currently, there is some discrepancy in the literature regarding the Entre Ríos lithologies. Some Authors [55,60,61] report loess sediments (Hernandarias Formation *c* and Tezanos Pintos units) only in the central and western section of the plain at the border of the Paraná Delta, with the eastern part dominated by fluvial/coastal sediments (Hernandarias Formation *b*). Others [59,76] extend loess units eastward to the Uruguay river. The 87Sr/86Sr ratios of the Hernandarias Formation *b* (0.70780 ± 0.00018, n = 13; Table 1, Fig 3) overlap substantially, given the high internal variability, with the loess of the Hernandarias Formation *c* (0.70760 ± 0.00007, n = 5) and the Tezanos Pintos units (0.70760 ± 0.00012, n = 8). This isotopic similarity suggests that loess sediments were either transported eastward across the lower Entre Ríos plain or homogenized by re-depositional processes.

The observed variations in Sr isotope signatures between Entre Ríos and Pampa plains likely reflect differences in the lithotypes eroded, and transported to the region by winds and rivers. Among the other components, the loess sediments outcropping in the area contain a high percentage of volcanic products (between 20–60%) related to Quaternary volcanism in the Andes [60,76–78]. Strontium isotope data from modern plants in this study are consistent with loess deposits at the same latitude, supporting the idea that volcanic contributions largely control their isotopic signature [78]. Throughout the Pleistocene and Holocene predominant winds have remained consistent (i.e., from SW to NE in the Pampa and from W to E in the Entre Ríos plain), although their intensity has weakened over time, reducing sediment deposition from S to N and from W to E [76,78]. Quaternary volcanism in southern Andes shows a rapid decrease in Sr isotopic signature between −32° and −34° from 0.706 down to 0.704 [78,79]. The lower isotopic values in Pampa may reflect the influence of more recent material eroded and transported by winds, while the decreasing trend in Sr isotopic values along the Pampa plain may be linked to the progressive weakening of wind transport. A similar process was proposed by Serna et al. (2020) [38] for the Rio Negro province, where bioavailable and inorganic Sr isotope values increase toward the east and north from the Andean arc due to the diminishing contribution of recent low-radiogenic volcanics.

In the Uruguay region, the sediments from the rivers show higher ^87^Sr/^86^Sr ratios (average of 0.70944 ± 0.00079, S.D. n = 5; Table 1; S1 File) compared to the outcropping lithologies (F. Dolores, F. Camacho, F. Fray Bentos and Cretaceous Formations, average of 0.70759 ± 0.00051, S.D. n = 12) possibly suggesting a contribution from enriched lithologies outside the studied area (e.g., the Precambrian granites in Fig 1).

### Sr isoscape and model consistency

**Fig 4:**
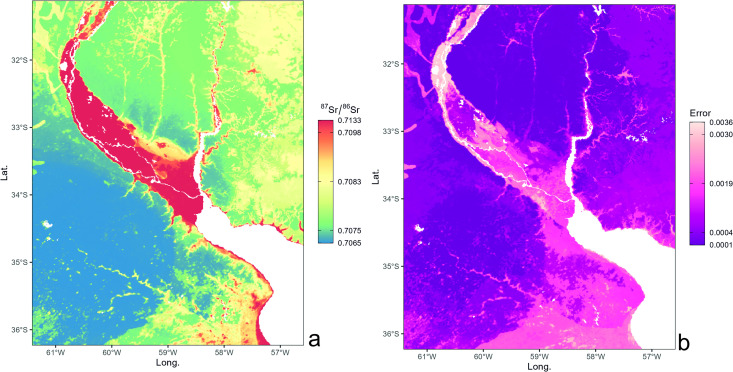
Spatial distribution of strontium isotopes in the Paraná Delta and surrounding areas. Isoscape RF model (a) and associated spatial uncertainty map (b).

The isoscape of bioavailable strontium in the Paraná Delta and surrounding areas, generated by the RF predictive model (Fig 4a), clearly distinguishes the more radiogenic areas within the Paraná Delta from the higher plains characterized by lower Sr isotope ratios. In Fig 4b, the prediction uncertainty map shows that the model’s predictive power is less accurate in the more radiogenic geological areas, registering an overall error range between 0.0001 and 0.0036. This has been observed before [18,42] and is consistent with the lithological complexity and wider (high) Sr isotope range of the Paraná Delta compared to other areas. The most plausible explanation is that elevated radiogenic values are in sharp contrast with those of less radiogenic mixing end-members such as aerosols, seawater, and precipitation, thereby increasing the observed Sr isotope variability of the bioavailable pool and reducing the accuracy of model predictions.

**Fig 5:**
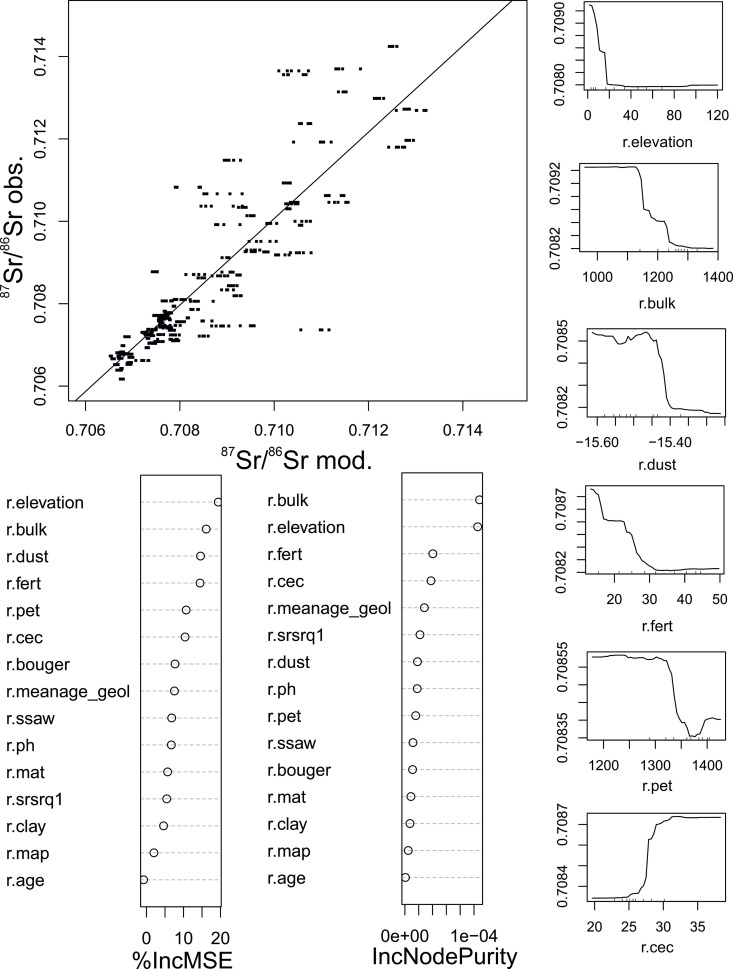
Predictive variables and model accuracy in Sr Isoscape analysis. Cross-validation scatterplots of observed vs. predicted values for the test–validation sample splits, Increase in Mean Squared Error (%IncMSE), Increase in Node Purity (IncNodePurity) and most predictive variables from Bataille et al. (2018; 2020) [18,43]: *r.elevation* is referred to SRTM topography (m), *r.bulk* is Bulk Density (kg*m^−3^), *r.fert* is Global Fertilization, *r.dust* is Multi-models average (g.m^−2^.yr^−1^), *r.pet* is Global Potential Evapo-Transpiration, *r.cec* is Cation Exchange Capacity. Other variables in Bataille et al. (2018; 2020) [18,43] and Reich et al. (2024) [71].

Fig 5 illustrates the cross-validation scatterplots of observed vs. predicted values for the test–validation sample splits. The scatterplot demonstrates a strong correlation between predicted and observed values, with a coefficient of determination (R²) of 0.82. This suggests that 82% of the variability in the observed values can be explained by the predicted values, suggesting a strong agreement between the two datasets.

The Root Mean Squared Error (RMSE) is 0.0008, indicating a relatively low average error between the predicted and observed values. This suggests that the predictive model used is accurate in forecasting the strontium isotopic ratio, with minimal deviations from the observed values. The deviations are determined by the higher isotopic variability in high-radiogenic areas, which results in greater prediction complexity. These results highlight that the Random Forest model employed is robust and provides reliable predictions, with an error level sufficiently low to be considered negligible in most practical applications. The RF error tends to underestimate the local isotopic variability observed in sites with high ^87^Sr/^86^Sr ratios.

The evaluation used the Percent Increase in Mean Squared Error (%IncMSE), which quantifies the reduction in model accuracy when a specific variable is randomly permuted. A higher %IncMSE value indicates significant predictive importance. The most predictive variables, according to %IncMSE (Fig 5), are ‘r.elevation’ (in meters) and ‘r.bulk’ (soil bulk density in kg m^−3^). The predictive power of the variables was also evaluated using the Increase in Node Purity (IncNodePurity), which measures the contribution of a variable to the purity of the nodes within the decision trees of the model. Variables with higher IncNodePurity values contribute more significantly at tree splitting, enhancing the model’s classification accuracy. Fig 5 shows that *r.elevation* and *r.bulk* soil also yield high IncNodePurity, reinforcing their importance. *r.elevation* emerged as the most predictive in %IncMSE, with high values in %IncMSE (~20%) and IncNodePurity (~0.9 × 10^−4^), highlighting its relevance in predicting the ^87^Sr/^86^Sr ratio. Similarly, *r.bulk* shows %IncMSE of ~16% and IncNodePurity ~1.0 × 10^−4^. Among other variables, *r.fert* (global nitrogen fertilization) exhibited significant values (~14% in %IncMSE and ~0.4 × 10^−4^ in IncNodePurity) while *r.dust* shows high %IncMSE (~15%) but very low IncNodePurity (~0.2 × 10^−4^, Fig 5). Although the data shows differences related to lithologies (as reported in paragraph 2), the model does not show lithology-driven variables as highly predictive. This discrepancy can be explained by the fact that the covariates here used only take into account few geological properties of the terrain, the most important being the age of the primary source rocks (see Bataille et al., 2020 [43]). In the studied area, mainly formed by Holocene to actual sedimentary deposits of different origin, these geological variables could not properly predict the geological distribution, as the geological units have the same ages and are a mix of sediments from rocks of different age and lithotype. For this reason, elevation becomes a predictive variable because it indirectly differentiates the type of sediments: fluvial and marine deposits of the Paraná Delta and the elevated plains with loessic/continental deposits.

Another aspect to consider is that the isotopic composition of bioavailable Sr can be influenced by atmospheric deposition (precipitation, marine aerosols, dust), the presence of exogenous surface deposits (such as loess, glacial till, cover sands, etc.), mixing processes between different strontium reservoirs, and anthropogenic influences such as fertilizer and air pollution [28,38]. These processes vary across different areas and can cause significant shifts in the bioavailable ^87^Sr/^86^Sr ratio compared to the expected values based on surface geology, although the bioavailable Sr isotopic signature is comparable with those of loess sediments at the same latitude [78] suggesting a small or nonexistent contamination of our samples from anthropogenic sources.

### A preliminary case study for model’s applications

The database of bioavailable Sr isotope ratios provided in this work shows high variability in the ^87^Sr/^86^Sr ratios highlighting its potential for investigating spatial and temporal migration of humans and animals in the region. This isotopic framework serves as a vital tool for addressing archaeological questions, particularly in reconstructing past human and animal mobility within complex geological settings.

During the Late Holocene, numerous pre-Columbian societies underwent complex processes of territorial expansion across South America. Among them, the Guaraní, originally from southwestern Amazonia, expanded widely across the region to the Río de la Plata estuary [80], influencing technological developments and subsistence strategies. The Guaraní expanded in a short time, extending over a large area of South America, reaching almost a subcontinental scale. In this context, their rapid expansion and large territorial coverage (1,500,000 km^2^). These Amazonian forager-horticulturalists migrated from southwestern Amazonia and appeared in the Upper Paraná–Uruguay basin around 500 CE, then following a migratory route along the Uruguay River they reached the Paraná Delta around 1300 CE [80]. It is likely that these canoe-based groups travelled down the Uruguay River, which they used as their main navigation route to connect various villages throughout the region. The expansion of these forager-horticulturalists during the Holocene brought significant changes in technology, social and political structures, as well as new landscape management practices. However, the reasons behind this expansion remain debated, with scholars proposing explanations linked to population growth, climatic change, and an expansionist ideology [81]. Archaeological records along the low Paraná wetland suggest significant changes in settlement patterns and subsistence [82–84].

**Fig 6:**
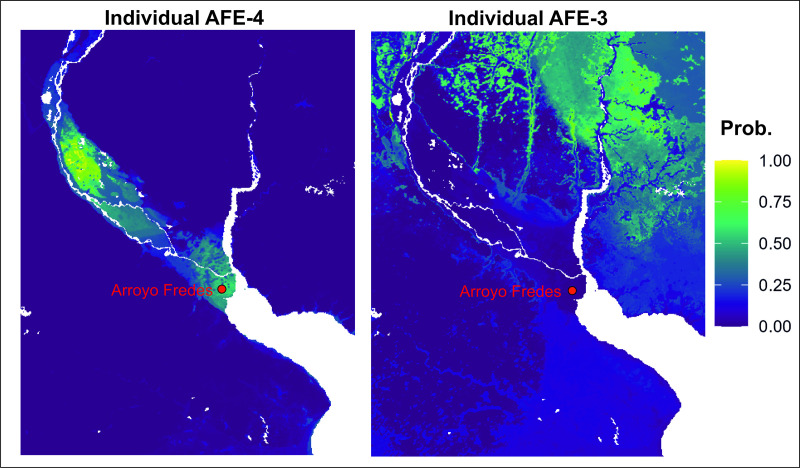
Estimating mobility for Arroyo Fredes individuals. Geographic origin probability estimated for two individuals from Arroyo Fredes, following the protocol described in Ma et al. (2020) [95]. Probability estimates (i.e., cell values) are scaled between 0 (low probability, purple) and 1 (high probability, yellow).

One of the best-known sites associated with Guaraní in the Paraná Delta is Arroyo Fredes, located at −34.1863° S and −58.5527° W. At the time of its occupation, the site was situated on a sedimentary island formed as a result of the progradation of the Paraná Delta and was likely 10–15 km from the Delta’s advancing front [85]. The site contains five radiocarbon dates ranging between 1229 and 1646 CE [85]. Several burials were recovered at Arroyo Fredes [86]. The ^87^Sr/^86^Sr ratios were measured in two individuals (S1 File). The first is an adult male (AFE-3), aged 20–30 years, and the second is a subadult (AFE-4) aged 3.5 years. Age-at-death was determined according to the protocols reported in Brooks and Suchey (1990) [87], Schmitt (2004) [88], Lovejoy (1985) [89], Meindl and Lovejoy (1985) [90], Acsàdi and Nemeskéri (1970) [91], AlQahtani et al. (2010) [92], and Maresh and Beal (1970) [93] while the classification of individuals into different age classes was performed following the Lewis method [94]. AFE-4 has an ^87^Sr/^86^Sr ratio of 0.71339 ± 0.00001, which falls within the local baseline range reported by Loponte et al. (2025) [67] (^87^Sr/^86^Sr range on local fauna = 0.71342–0.71377; S1 File). On the other hand, AFE-3 has an ^87^Sr/^86^Sr ratio of 0.70810 ± 0.00002, suggesting a non-local origin. We assessed the Sr isoscape for the provenance of the two individuals using a Bayesian probabilistic approach through the *assignR* package [95], which generates a probability density map indicating the likely provenance of each sample (Fig 6). As expected, AFE-4 shows high compatibility with the Paraná Delta region, whereas AFE-3 appears to have migrated from the north along the Uruguay River (Fig 6). These results align with the discovery of widespread Guaraní archaeological sites along the Uruguay River and support the widely accepted theory that this population expanded southward along South American river systems [96]. The Sr isoscape model presented in this study offers a valuable opportunity to trace Guaraní migration routes along the rivers spanning from southwestern Amazonia to the Río de la Plata estuary [97,98]. This pioneering contribution, relevant not only to the immediate region but to the broader La Plata Basin, offers an important resource for investigating other populations that inhabited these areas across different historical periods. This example illustrates the potential of the model, emphasizing its usefulness as a powerful tool for environmental, economic, and archaeological studies. The model’s efficacy could be further enhanced by integrating it with another stable isotope systematics.

## Conclusion

This study presents a comprehensive dataset of bioavailable ^87^Sr/^86^Sr ratios from the Paraná Delta area and surrounding high plains, revealing significant heterogeneity driven by sediment composition and, in turn, linked to the isotopic signatures of their geological source. The observed ^87^Sr/^86^Sr ratios in the terrestrial biosphere align with the lithological data, underscoring the strong influence of surface composition and sedimentary processes in shaping isotopic variability. This study highlights the importance of considering lithological environments and the age of rocks when interpreting Sr isotope signatures in sedimentary environments.

A high-resolution spatial distribution map of bioavailable Sr for the La Plata basin region was developed using advanced modeling methods, i.e., the Random Forest model. This predictive framework provides a detailed depiction of the ^87^Sr/^86^Sr variability across northeast Argentina and southwest Uruguay, offering a valuable baseline for provenance, environmental and forensic studies.

The ^87^Sr/^86^Sr analysis at the Arroyo Fredes site provides a simple case study for possible applications of this isoscape. The isotopic data suggest one individual as having a possible local origin and another having a non-local origin, in agreement with the theory of Guaraní migration along the Uruguay River into the Paranà Delta around 1300 CE. This model enhances our interpretative capabilities by facilitating precise identification of migration patterns and settlements origins.

The strontium isoscape map developed here constitutes a powerful tool for analyzing various aspects of pre-Columbian populations, including patterns of mobility and migration, resource exploitation, social interactions, and the provenance of prey. By facilitating a deeper understanding of past human and animal movements, this model will enhance our ability to reconstruct prehistoric socio-economic networks in the region.

While this study has successfully developed a precise bioavailable Sr isoscape for the region, further research is necessary to expand and refine its applicability. Increasing the dataset with additional sampling points and extending the geographic area of interest would enhance the predictive accuracy of the model. Future efforts should also focus on integrating this dataset with archaeological and ecological research to explore broader questions related to ancient land use, trade networks, and environmental adaptations.

In conclusion, this research provides essential baseline data for strontium isotope research in South America, offering a robust foundation for future archaeological, ecological and forensic provenance studies. By enhancing our understanding of ancient mobility and resource circulation, this study will contribute to the broader reconstruction of past human-environment interactions in the region.

## Supporting information

S1 File ^87^Sr/^86^Sr variability: data for RF model refinement. ^87^Sr/^86^Sr values of modern samples analysed in this study with geographical and geological information and literature values of bioavailable Sr utilised to refine the RF model.
(XLSX)

S2 File Analytical session data. ^87^Sr/^86^Sr values of reference material NBS987 during the analytical sessions.
(XLSX)

S1 Fig External predictor’s correlations. Correlation of the 24 external predictors from Bataille et al. (2020) [43] and Reich et al. (2024) [71] calculated as R (version 4.0.5, available at R core Team 2024).
(TIF)

S3 File Inclusivity-in-global-research-questionnaire. (DOCX)
