# Supplementary figures and images for: Random forest-based bioavailable strontium isoscape for environmental and archaeological applications in central eastern Argentina and western Uruguay
Source: PLoS One. 2025 Jul 15;20(7):e0326047. doi: 10.1371/journal.pone.0326047 (PMC12262893; doi:10.1371/journal.pone.0326047)

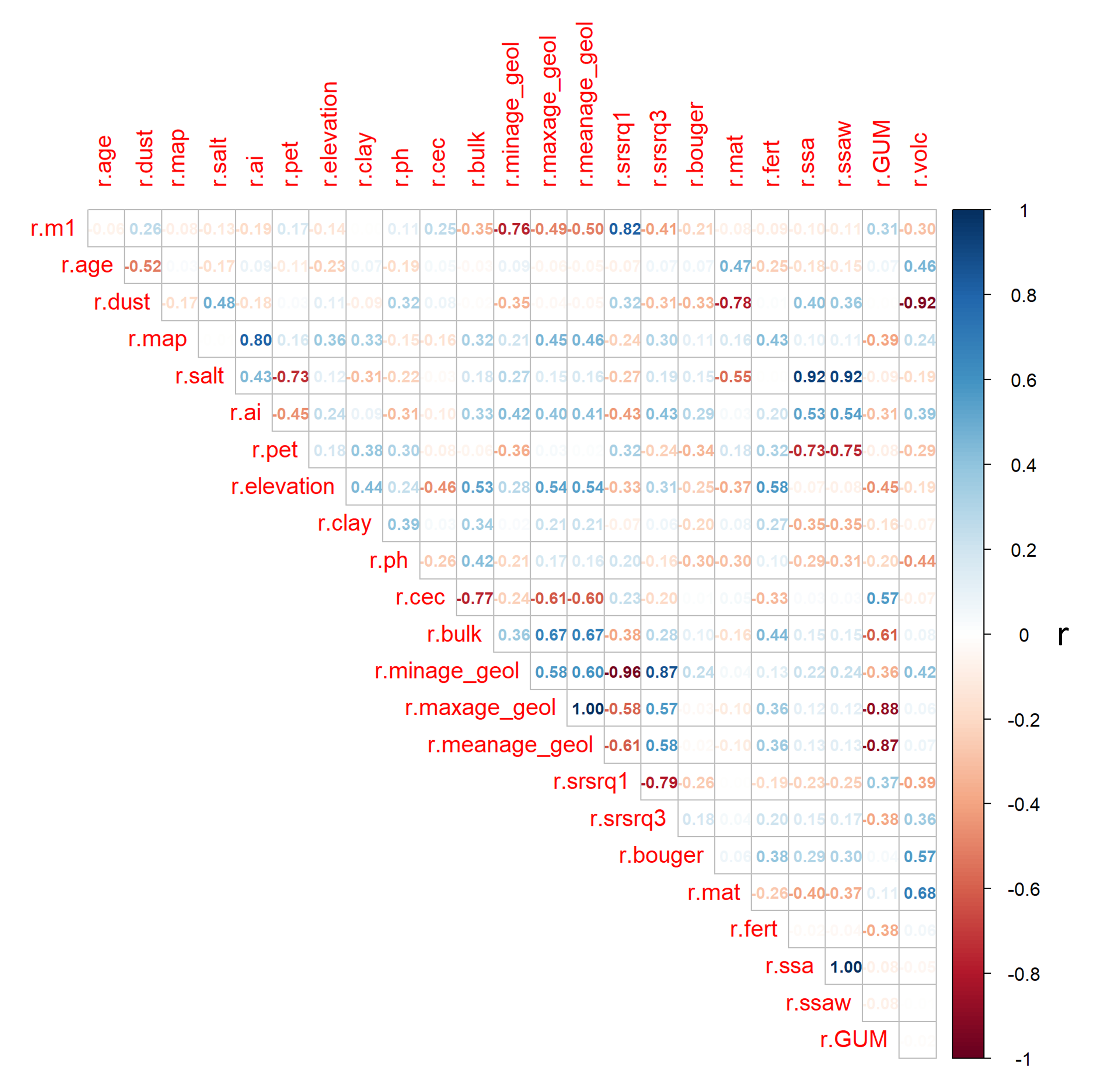

Supplement: S1 Fig — Correlation of the 24 external predictors from Bataille et al. (2020) [43] and Reich et al. (2024) [71] calculated as R (version 4.0.5, available at R core Team 2024). (TIF) [file pone.0326047.s003.tif]
